# PolyQ Length of the Clock Gene Is Correlated With Pelagic Larval Duration in the Damselfishes (Pomacentridae), but Within a Species Habitat Availability Counts

**DOI:** 10.1002/ece3.71259

**Published:** 2025-04-25

**Authors:** Gregor Schalm, Simon Kaefer, Philipp Krämer, Anna‐Lena Jäger, Michael J. Kingsford, Gabriele Gerlach

**Affiliations:** ^1^ Research Group of Animal Biodiversity and Evolutionary Biology Carl‐von‐Ossietzky‐University of Oldenburg Oldenburg Germany; ^2^ Marine Biology and Aquaculture College of Science and Engineering, James Cook University Townsville Australia

**Keywords:** coral reef fish, larval dispersal, migration, otolith, settlement

## Abstract

Immediately after hatching, larvae of coral reef fish leave their natal reef environment and begin their pelagic larval phase probably to avoid high predation on the reef. The time they spend in the open ocean (pelagic larval duration, PLD), before settlement varies from species to species and depends partly on developmental processes that eventually require re‐settlement to a reef. The polyglutamine region (PolyQ) as part of the clock gene has been suggested as a possible candidate that could control developmental processes and potentially the time until settlement, which can be determined by counting the rings of the otoliths. We studied the potential relationship between the number of glutamine repeats in 20 species of pomacentrids and their PLDs. Most pomacentrids came from similar locations, so we avoided the impact of latitudinal clines on PLD. Within the *clock* gene, we found two main distinguishable, variable glutamine‐rich regions (PolyQ and Qrich). Considering phylogenetic relationships, PolyQ/Qrich repeat length and pelagic larval duration were significantly positively correlated. However, when analyzing this relationship in a single species, the neon damselfish ( *Pomacentrus coelestis* ), we did not find a significant correlation between PolyQ length variation and PLD. Instead, we found a significant reduction of PLD in years with increased habitat availability. Our results show that glutamine‐rich regions can influence the timing of settlement on a broader scale, but that ecological factors—such as habitat availability—can also have a significant impact.

## Graphical abstract

The length of a part of the ‘clock’ gene explains the duration of dispersal in coral reef fishes. However, ecological factors such as the availability of habitats can override this effect.

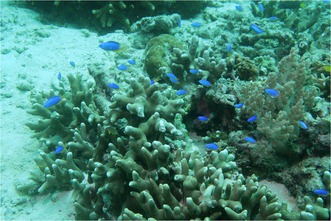

## 1 Introduction

Coral reef fish produce thousands of larvae, which are transported to the open ocean immediately after hatching. The time they spend in the open ocean (pelagic larval duration, PLD) depends on the species and ranges from a few days to several weeks (Brothers et al. 1983; Thresher et al. 1989; Wellington and Victor 1989; Wilson and McCormick 1999; Bay et al. 2006; Kingsford et al. 2011). In the first phase of PLD, the larvae are regarded as plankton that are distributed by the current (Leis and McCormick 2002), before they develop into more efficient swimmers (post‐flexion stage) (Downie et al. 2020) that have multiple sensory orientation abilities which can guide them back to a reef for settlement (Kingsford et al. 2002; Gerlach et al. 2007). Scheltema (1967) developed the ‘critical size hypothesis’ proposing that pelagic larvae (vertebrate or invertebrate) must reach a critical size to begin metamorphosis and settle to their suitable benthic habitat. Testing this hypothesis, Kingsford et al. (2022) showed that for the neon damselfish ( *Pomacentrus coelestis* ) variation in age at settlement was greater than that of size at settlement for fish collected in waters of different temperatures. They concluded that for settlement fish need to reach a specific size which varied with water temperature and food. Other factors such as oceanography and settlement site selection have been used as potential explanations for the 55%–60% variation in PLD (Kingsford et al. 2022). Temperature in particular leads to fluctuations in the developmental speed of the fish from the embryonic stage onwards (Johnston 2006); higher temperature mostly accelerates development. Therefore, near the equator, PLDs of the same species are often shorter than at higher latitudes where water temperature is lower (Bay et al. 2006; Sponaugle et al. 2006).

But developmental processes such as growth are also subject to genetic control. One important and well‐studied timing mechanism—which is ubiquitous from bacteria to animals—is the circadian clock. It is controlled by highly conserved genes known as “clock genes”, which have been shown to control the rhythmicity of physiology and behaviour in the 24‐h cycle (Mazzoccoli et al. 2012), but also over longer periods of time [see overview Le Clercq et al. (2023)].

The poly‐glutamine repeat (PolyQ) is one important regulative component of the circadian clock that can fine‐tune the timing of developmental transitions according to the environment (Kyriacou et al. 2008). It is known from studies of glutamine repeats in the yeast transcriptional regulator Ssn6 (Cyc8) that PolyQ influences the solubility and thus the activity of the transcribed protein, resulting in target gene expression that is dependent on the repeat length (Gemayel et al. 2015). To our knowledge, a direct effect of PolyQ on the growth of organisms has not been shown so far. Previous observations of length polymorphisms in the PolyQ region in model species such as the Chinook salmon ( *Oncorhynchus tshawytscha* ) have found evidence of selection for specific variants along a latitudinal gradient (O'Malley and Banks 2008; O'Malley et al. 2010). As photoperiod is tied to latitude, this partially explains how environmental changes modulate the circadian clock on a circannual basis, possibly driving the timing and duration of migration.

A significant association between PolyQ variability and factors contributing to migration patterns, annual synchronicity in life events, or geographical processes has been illustrated in multiple species‐specific studies, while associations were not clear or absent for other lineages [see for review: (Le Clercq et al. 2023)].

Our aim was to further investigate the potential interaction of PolyQ and dispersal duration in coral reef fishes, especially pomacentrids; here the duration of the dispersal can be determined very precisely on the basis of the daily formed rings of otoliths, which provide a comprehensive database for the analysis of a possible relationship between PolyQ and PLD of different species. While almost all pomacentrids have a dispersal stage, their PLD varies greatly, even if they originate from the same area (Ronald et al. 1989)—allowing exploration of the genetic basis of PLD without the interference of latitudinal clines.

We studied the impact of variation of PolyQ on PLD at the individual level of the species *Pomacentrus coelestis* using our sample collection from different years (Gerlach et al. 2007, 2021; Kingsford et al. 2014, 2017). This species is a short‐living coral reef fish with a maximum age between 127 and 160 days (Kingsford et al. 2017). *P. coelestis* is most abundant in coral rubble, especially on perturbed reefs (Gerlach et al. 2021) and on outer reef slopes (Doherty et al. 1996). The Great Barrier Reef was hit by the tropical cyclone Hamish in 2009 (Woolsey et al. 2012). Due to this event, the live coral cover dropped to less than 20% on many reefs, offering additional habitat for *P. coelestis* since this fish is the first coloniser of disturbed reefs (Gerlach et al. 2021). This allowed us to study the potential influence of PolyQ on PLD in two different situations, high and low habitat availability, at four adjacent reefs. Our phylogenetic approach, together with a detailed species‐specific genetic and PLD analyses including a natural change in habitat availability and previous results (Kingsford et al. 2017, 2022) should contribute to a holistic understanding of the interplay between genetic and ecological effects on PLD and settlement in coral reef fishes.

In this study, our aims were: (1) to characterise the PolyQ domain of the *clock* gene in coral reef fishes; (2) to determine the relationship between PolyQ length and PLD among 20 pomacentrid species; and (3) within a single pomacentrid species, *P. coelestis* , considering PLD variations and the role of habitat availability in this species. Therefore, we first sequenced PolyQ in multiple pomacentrid species to understand its general structure. Using this information, together with the available data of PLD in the literature, we analysed their possible correlation on a species level. In the next step, we investigated the role of PolyQ in *P. coelestis* by measuring the PLD for each individual using otoliths and the PolyQ length via fragment length analysis. As a last step, we took advantage of the fact that habitat availability changed significantly during the sampling of *P. coelestis* , allowing us to determine its effects on PLD.

## 2 Materials and Methods

### 2.1 Sampling and Determination of Coral Cover

**FIGURE 1:**
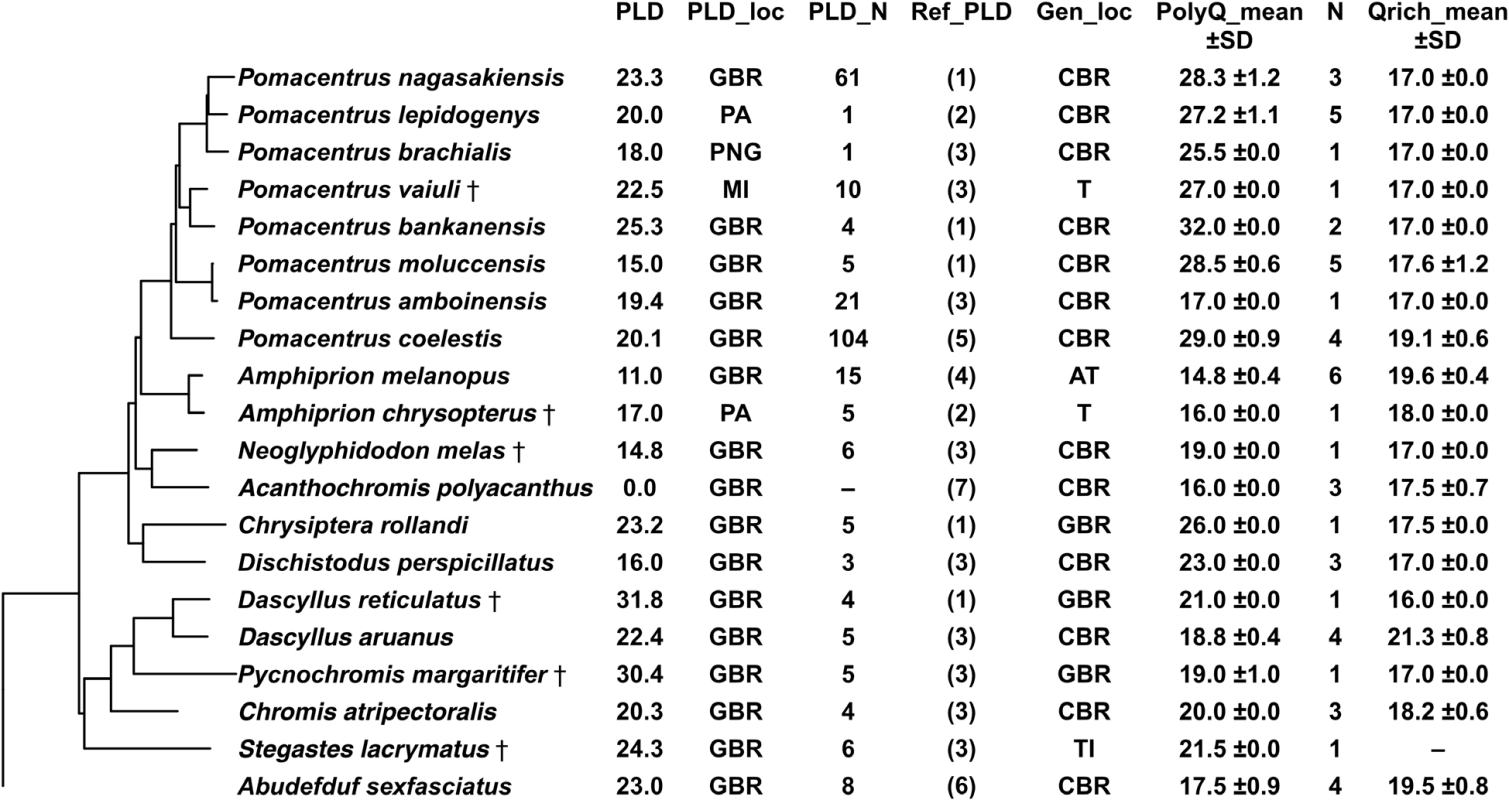
Phylogeny of 20 species of Pomacentridae based on McCord et al. (2021) and information on PLD and PolyQ and Qrich fragment lengths. PLD: Pelagic larval duration (days) PLD_loc: Location where fish were sampled (GBR: Great Barrier Reef, PA: Palau, PNG: Papua New Guinea); PLD_N: Number of fish for which PLD was determined; Ref_PLD: References for PLD: (1) (Wilson and McCormick 1999); (2) (Wellington and Victor 1989), (3) (Thresher et al. 1989) (4) (Bay et al. 2006) (5) (Kingsford et al. 2011) (6) (Brothers et al. 1983) (7) (Robertson 1973) Gen_loc: Sampling location of fish for which the number of PolyQ and Qrich was determined (CBR: Capricorn Bunker Reef Group which is located at the southern tip of the GBR, T: Tonga, TI: Timor, AT: Aquarium trade, † Museum sample); PolyQ_mean: Mean length of PolyQ ±SD (standard deviation); *N*: Number of fish for which PolyQ and Qrich was determined; Qrich_mean: Mean length of Qrich ±SD.

We analysed 20 damselfish species for PolyQ and Qrich variability (Figure 1); the majority (*n* = 16) of which originated from the Great Barrier Reef (GBR), Australia. One species ( *Amphiprion melanopus* ) was derived from aquarium trade (AT), two individuals of two species each originated from Tonga ( *Pomacentrus vaiuli* and *Amphiprion chrysopterus* ) and one (*Stegastes lacrymatus*) from Timor‐Leste (TI). Per species, we used 1–6 individuals.

The intraspecies variation of PolyQ length, PLD, and the impact of habitat availability were assessed by analysing the neon damselfish *P. coelestis* . Individuals were collected always in January in different years and at different reefs One Tree Island (OTI), Fitzroy, Heron, and Lamont all are reefs of the Capricorn Bunker Reef group (CBR), southern Great Barrier Reef [GBR, for more details see (Gerlach et al. 2021)] before and after the category 4–5 cyclone Hamish hit the CBR in 2009, destroying live coral cover and providing additional settlement habitat for *P. coelestis* . Coral cover was recorded annually by us at One Tree Island from 2007 at a site on the North East slope (Site 1, 23_29.708 S, 152_05.887E). A replicate site 2, about 2 km from site 1, was monitored approximately biennially from 2010 (Site 2: 23_29.214 S, 152_05.498E). At each site, percentage cover was estimated along five transects (5 × 25 m). This sample size gave a 95% chance of detecting any type of substratum that represented 10% or more of cover (Kingsford and Battershill 1998). Public domain data from the Australian Institute of Marine Science (AIMS) long‐term monitoring provided benthic cover data at three sites on the exposed side (facing east) of Broomfield and Lady Musgrave reefs annually; five transects were sampled at each site per reef (6–9 m deep). Data on total hard coral cover include all coral morphologies, and tabulate acroporids, which are a major habitat‐forming coral, especially on the exposed sides of the CBR (Woolsey et al. 2012).

### 2.2 Genetic Analysis for Species Identification and Analysis of *Clock* Fragments Using Sanger Sequencing

Samples were sequenced for *cytochrome‐c‐oxidase subunit 1* (COI) to verify species and for one to two areas within the *clock* gene. DNA was extracted from a pinpoint‐sized part of tissue using Chelex 100 Resin (5% w/v) solution (Bio‐Rad) by embedding the samples into Chelex beads and incubating for 20–30 min at 95°C, spinning down and storing until further processing at room temperature.

PCR was conducted using FastStart High Fidelity PCR System (Roche) following manufacturer's instructions. The final concentration within the reaction mix was 1% DMSO, 1x FastStart buffer with 1.8 mM MgCl_2_, 0.5 μM of both forward and reverse primer, 200 μM dNTP and 0.05 U/μl Taq and 5% (v/v) DNA. 38 cycles were performed with an annealing temperature of 54°C. We used the FishF1 and FishR2 primer from Ward et al. (2005) elongation time was 90 s. Samples were cleaned up using magnetic beads (HighPrep PCR Clean Up System, MagBio) following manufacturer's instructions and send to sequencing service (Macrogen Europe B.V. or GATC Biotech) with the forward primer.

Species were identified using the Barcode of Life Data (BOLD) system (Ratnasingham and Hebert 2007) and its Species Level Barcode Records. Species were identified based on the “Best ID” output of BOLD. *Acanthochromis polyacanthus* had the lowest similarity and matched only with 94.5% to 94.8% and was assigned manually. It was not possible to distinguish sequences between *Amphiprion melanopus* and *A. ephippium* based on COI sequences due to high similarity. For this reason, we used the morphological characteristics of the phenotype to identify the species.

**FIGURE 2:**
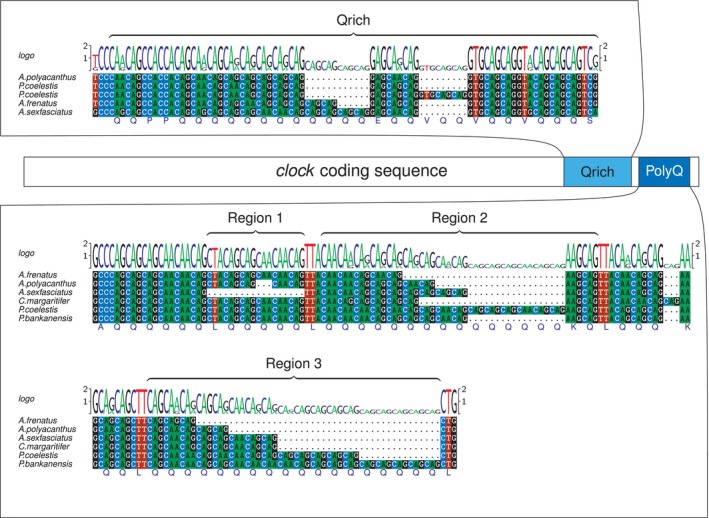
Overview of the sequenced sections of the clock gene containing Qrich and PolyQ, the latter consisting of three regions in six species *A. frenatus*: *Amphiprion frenatus* , *A. polyacanthus*: *Acanthochromis polyacanthus* , *A_sexfasciatus* *Abudefduf sexfasciatus* , *P. margaritifer*: *Pycnochromis margaritifer*, *P. coelestis*: *Pomacentrus coelestis* , *P. bankanensis*: *Pomacentrus bankanensis* . A representative subset of sequences from different pomacentrid species is shown using the texshade package (Beitz 2000) for LaTeX. The size of the blue bars (Qrich and PolyQ) refers to the size of the sequenced sections in relation to the known clock coding sequence. Alignments show parts of the sequences containing glutamine repeats. The nucleotides are colour coded (red = thymine, green = adenine, black = guanine and blue = cytosine) and size of letters represents frequency within the logo line. Below the alignment the amino acid sequence is shown using one letter code. Qrich is characterised by interrupted glutamine repeats, whereas PolyQ has 3 distinguishable, almost pure regions, which are separated by smaller segments. *Pycnochromis margaritifer* was the only species with an additional glutamine between region 1 and 2. Region 1 of PolyQ was almost identical for all species; differences were species‐specific and were also observed in *Abudefduf sexfasciatus* and the non‐dispersing *Acanthochromis polyacanthus* , only the former lacked a CTA (leucine).

To get first insight into the variability of *clock* gene structure, we sequenced the c‐terminal, glutamine‐rich area of the *clock* gene (Darlington et al. 1998; Schalm et al. 2021). We sequenced two fragments separately. In the following, we refer to the first fragment as “Qrich” and to the second one as “PolyQ” (Figure 2).

We used the same PCR protocol described for COI, but with an annealing temperature of 58 C. We sanger sequenced the amplicons with forward and reverse primers (Table 1) at GATC and manually using the Beckman Coulter CEQ 8000 platform, adhering to standard protocols.

**TABLE 1:** Overview of primer sequences used for sequencing (seq) and fragment length (MS) analysis.

Primer	Locus	Analysis	Sequence
Pom Qrich seq fwd	Qrich	Sequencing	AGATAGGAGTACACCCGGCA
Pom Qrich seq rev			CCTGCAGGAACTGTGTTTGC
Pom Qrich MS fwd	Qrich	Fragment length	M13CAGCTGCTCACCAAGCTAGT
Pom Qrich MS rev			CCTGCAGGAACTGTGTTTGC
Pom PolyQ seq fwd	PolyQ	Sequencing	CCACCCAGTTGATCCTGCAA
Pom PolyQ seq rev			AACAATCCTCTGGCGGCTC
Pom PolyQ MS fwd	PolyQ	Fragment length	M13CCACCCAGTTGATCCTGCAA
Pom PolyQ MS rev			CAACTCCACTTCAGCCAGCT

*Note:* Primers were established using Primer3 (Untergasser et al. 2012) and Primer‐BLAST (Ye et al. 2012). Forward primer are labelled with the M13‐tag to be used with fluorescent labelled primer (Schuelke 2000).

Sequences of heterozygote samples were reconstructed by a combined approach using Mixed Sequence Reader (Chang et al. 2012), Indelligent v1.2 (Dmitriev and Rakitov 2008), Champuru 1.0 (Flot 2007) (all with standard settings) and manual evaluation. After we could resolve the sequence *in silico*, we cross‐checked the overall sequence length using fragment length analysis. Allele a refers to the shorter, and allele b refers to the longer of both alleles. Sequences were aligned using Ugene v36.0 (Okonechnikov et al. 2012) and MUSCLE (Edgar 2004) and we manually improved the alignment within the glutamine repeats.

We determined one region of Qrich (Figure 2), but subdivided the PolyQ fragment into three different regions separated by intermediate sequences for further analysis. Based on this, we sequenced all 61 samples of 20 species for PolyQ and Qrich using Sanger sequencing.

After we verified by sequencing that different alleles of PolyQ and Qrich in different pomacentrid species were based on different numbers of repeats and not on changes in the flanking region or indels, we determined the length of PolyQ and Qrich for *P. coelestis* using capillary sequencing.

Both *clock* fragments were amplified using HotStarTaq (Qiagen) and labelled with universal fluorescent primer (M13‐tags with D2‐PA, D3‐PA, D4‐PA dyes, Sigma‐Aldrich/Beckman‐Coulter) (Schuelke 2000). The reaction mix contained in a 10 μL reaction at a final concentration: 1x Q‐solution, 1x CoralLoad PCR Buffer, 5 mM MgCl, 0.25 mM dNTP, 0.1 μM forward and 0.25 μM reverse and labelled universal primer, 0.025 U/μl HotStarTaq DNA Polymerase and 10% sample DNA. The cycling protocol was adapted for the use of universal primers (Gerlach et al. 2016). After activation of the HotStarTaq DNA Polymerase (95°C, 15 min), the fragment was initially amplified for 20 cycles (denaturation: 94°C for 30 s, annealing: 60°C for 45 s, elongation: 72°C for 45 s). Thereafter, another 20 cycles were run with a lower annealing temperature (53°C) to ensure that the labelled primers were incorporated. After final elongation (72°C for 10 min) samples were stored at 4°C.

The solution of PCR‐fragments of different samples was pooled (for PolyQ: 50% D2‐PA, 30% D3‐PA and 20% D4‐PA labelled, for Qrich: no D3‐PA‐labelled PCR was conducted due to insufficient fluorescence) and diluted 1:30 (v/v) with sample loading solution containing Sciex DNA Size Standard Kit 400 BP 1:80 (v/v). Length was determined using capillary sequencing (Beckman CEQ 8000 Genetic analysis system) with the Frag‐4 method.

**FIGURE 3:**
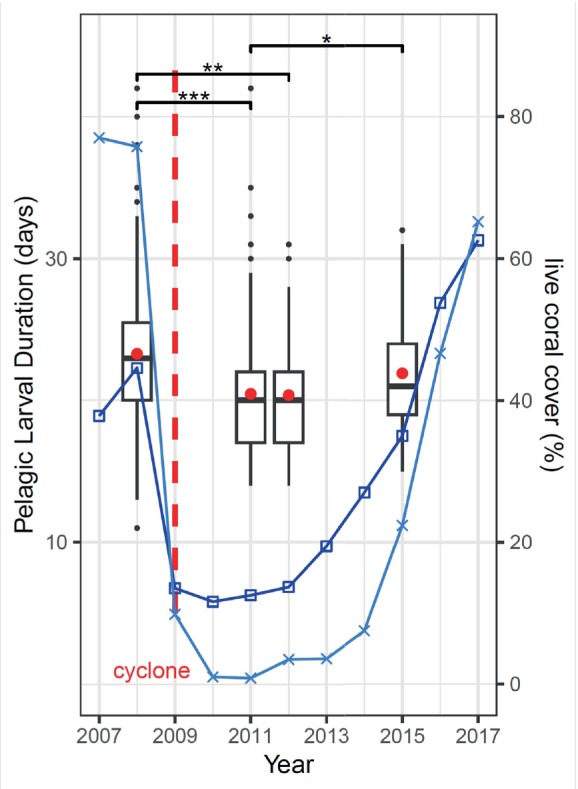
Pelagic larval duration of *Pomacentrus coelestis* as boxplots in four different years. The mean PLD of the respective years is shown as red dots. In 2009, tropical cyclone Hamish (indicated by the dashed red line) hit the Great Barrier Reef causing high damage on corals, shown as live coral cover of the Broomfield Reef (blue line with □) and Lady Musgrave Reef (light blue with ×) based on Gerlach et al. (2021). Low coral cover means high settlement habitat for *Pomacentrus coelestis* . *p < 0.05, **p < 0.01, ***p < 0.001; black dots are outliers (> 2 SD). Number of fish used for otolith analyses: 2008 *n* = 159, 2011 *n* = 127; 2012 *n* = 25; 2015 *n* = 74 (Table S2).

Data were analysed using the CEQ 8000 Genetic Analysis System 9.0.25 (Beckman Coulter). Peaks were scored by eye from electropherograms and used to calculate the actual glutamine repeats. The length of the flanking regions were defined from the sequences of three sequenced *P. coelestis* (Figure 3) in Qrich (230 bp) or PolyQ (156 bp) and was subtracted from the measured fragment length and divided by three (for the triplet code). The resulting number was rounded to whole numbers. To determine the impact of PolyQ and Qrich on PLD we used the longest, shortest, and the mean value of the glutamine region of both alleles per individual.

### 2.3 Determination of Pelagic Larval Duration (PLD) of *P. coelestis* and Other Pomacentrid Species

We determined the PLD of *P. coelestis* following the protocol of Kingsford et al. (2011). A randomly selected sagittal otolith was extracted and attached to the edge of a glass slide with Crystalbond 509–3 thermoplastic glue and a lighter, with the distal end extending beyond the slide. The otolith was ground to the primordium (nucleus) using wet sandpaper (P 3000) and repositioned with the flat side facing down on the slide. The protruding side was also ground to the primordium. When rings were countable, a whitening toothpaste was used for polishing (Colgate Maxwhite, high RDA value >80). Digital pictures were taken using a Leica DMLB light microscope and a Sony α 6500 with the Leica 541,007 adapter. The rings were counted from the primordium to the settlement mark after which the ring width decreased significantly (Figure S1). PLD increments generally start in otoliths at or very close to hatching—not immediately post fertilisation. The duration of egg brooding in tropical pomacentridae is 4–5 days (where 8 day is mentioned the fish are often form high latitude), but the otic capsule only forms late in development.

From previous studies, we used PLD data from otolith analyses of other pomacentrid species, obtained with similar methods as described above, to investigate potential correlation of glutamine repeats and PLD (Figure 1). We used information about PLD of fishes (whenever available) from GBR (ideally near the CBR) to avoid variation based on different sampling locations. If various PLDs were described for the same species, we used PLD determined by the highest number of individuals.

### 2.4 Statistical Analysis

Taxa sharing a larger fraction of their evolutionary history (i.e., having a more recent common ancestor) are likely to be more similar to one another (even after considering the effects of various potential predictors) leading to non‐independent residuals (Mundry 2014). The method of phylogenetic generalised least squares (PGLS) (Grafen 1989) has been developed to cope with such phylogenetically driven non‐independent residuals. We used the PGLS model (pgls, caper package for R (Orme 2011) to investigate the linkage between the number of glutamine repeats of PolyQ/Qrich and PLD. Lambda, kappa, and delta branch length transformations were optimised by maximum likelihood. Branch length transformations: kappa (ML): 3.000, lower bound: 0.000, *p* = 0.044, upper bound: 3.000, *p* = 1, 95.0% CI: (0.099, NA); lambda (ML): 0.255, lower bound: 0.000, *p* = 0.003, upper bound: 1.000, *p* = 0.0003, 95.0% CI: (0.002, 0.768); delta (ML): 0.185, lower bound: 0.000, *p* = 1.5e‐06, upper bound: 3.000, *p* = 7.46e‐0, 95.0% CI: (0.016–0.610).

A general linear model (GLM) was used to analyse the impact of PolyQ and Qrich on PLD in *P. coelestis* . Data were analysed with R (version 4.4.2). Diagrams were prepared using ggplot2 (Wickham 2009). To understand the impact of habitat availability on PLD, we tested for differences in PLD for the 4 years using a Kruskal‐Wallis test (Hothorn et al. 2008). To pinpoint significant between‐year differences, we carried out pairwise Dunn tests with Bonferroni correction for multiple testing using rstatix.

## 3 Results

### 3.1 Clock Gene Associated Number of Glutamine Repeats in 20 Pomacentrid Species and Its Correlation With Pelagic Larval Duration (PLD)

We successfully sequenced the glutamine repeat in 20 pomacentrid species (Figure 1) to analyse for possible correlations between the number of glutamine repeats and PLD. We found two gene segments, Qrich and PolyQ, containing glutamine repeats, which are located at the c‐terminal end of the *clock* gene (Figure 2). We assume that both fragments are located on different exons, as amplification of both fragments on one amplicon was successful in an exemplary experiment using *A. ocellaris* mRNA derived cDNA (data not shown), but was repeatedly not successful using gDNA. Both, but especially PolyQ, showed high variation in length. We could not successfully sequence Qrich in *Plectroglyphidodon lacrymatus* . Qrich contained between 16 and 22 glutamine repeats, interrupted by various amino acids (glutamic acid, proline, valine). Occasionally, one additional valine was found (Figure 2).

For better comparison, we separated PolyQ into three different regions, which differed largely in the number of glutamine repeats. Region 1 differed in only two species: *Acanthochromis polyacanthus* had one less glutamine than all other species, and *Abudefduf sexfasciatus* lacked the entire region (Figures 2 and 3). Region 2 was the second largest region and contained between 6 and 16 glutamine repeats with a lysine before the last glutamine in all but 3 samples. Between Region 2 and Region 3, six amino acids were enclosed by a leucine at the start and the end. In one sample of *Pycnochromis margaritifer*, we found an additional glutamine between these two regions. Region 3 contained between 3 and 18 glutamines (Figure 2). In total, the entire PolyQ region contained between 13 and 32 glutamines.

Using this genetic information of the combined three PolyQ regions and Qrich fragment lengths and literature data about the PLD, we tested for a potential correlation. PGLS was used to relate both PolyQ and Qrich length to PLD (Smaers et al. 2016) (Figure 1). The phylogenetic relationships for these calculations were obtained by pruning the tree from McCord et al. (2021) to our used species subset. The phylogenetic signal for each gene was measured using the R package phytools 0.7–90 (Revell 2012).

The phylogenetic generalised linear model revealed a significant influence of the total PolyQ (*p* = 0.0397) and of the Qrich (*p* = 0.0307) on the PLD (Model overall F‐statistic = 7.25, df = 2, 15, adjusted *R* ^2^ = 0.42, *p* = 0.006) (Table 2). The *R* ^2^ value in the context of a PGLS model indicates how well the model explains the variation in the dependent variable after accounting for the phylogenetic relationships among the species. It provides a measure of the proportion of variance in the dependent variable (PLD) that is predictable from the independent variables, adjusted for phylogenetic non‐independence.

**TABLE 2:** Summary of results of phylogenetic generalised least squares (PGLS) regression analysis for PolyQ and Qrich alleles in the studied fish species.

	Estimate	Std. error	*t*	*p*
Intercept	40.45	13.15	3.08	0.0077
PolyQ	0.418	0.19	2.25	0.0397
Qrich	−1.49	0.62	−2.38	0.0307

*Note:* The phylogenetic tree used as input for PGLS was retrieved from McCord et al. (2021). Phylogenetic generalised least squares (PGLS) models were fitted for the full list of 18 species for which the most common allele and migration data were available. The test was run using the R package caper 1.0.1 (Orme 2011). *Acanthochromis polyacanthus* and *Stegastes lacrymatus* were excluded since *A. polyacanthus* has no PLD and we could not determine Qrich in *S. lacrymatus*. Coefficients: Residual standard error: 10.39 on 15 df, Multiple R‐squared: 0.492, Adjusted *R* ^2^: 0.42, *F*‐statistic: 7.25 on 2 and 15 df, *p*‐value: 0.0063.

We excluded two species from the statistical analysis: *Stegastes lacrymatus*, because we could not amplify a Qrich fragment, and *Acanthochromis polyacanthus* , because they have no dispersal phase (Robertson 1973) and therefore have a qualitatively different phenotype. Nonetheless, the PolyQ and Qrich sequences of *A. polyacanthus* did not reflect this lack of dispersal; the lengths of both fragments were well within the range of the other species (Figure 2) with a mean length of PolyQ of 16 (others 14–32) and of Qrich of 17.5 (others 16–21.25).

### 3.2 *P. coelestis* Settled Earlier When More Habitat Was Available

To understand whether the number of glutamine repeats could be responsible for the variation in PLD within a species, we determined the PLD of 156 individual *P. coelestis* and their PolyQ and Qrich lengths (Table S1 and Figure S2). Overall, we did not find a significant correlation of PolyQ/Qrich length and PLD using a linear model (ordinary least squares) (PolyQ: Estimate = −0.029, Std. Error (SE) = 0.039, *t* = −0.734, *p* = 0.464 Qrich: Estimate = −0.012, SE = 0.051, *t* = −0.228, *p* = 0.82) (see Figure S2).

*P. coelestis* are the first fish colonizing destroyed reef habitats. To understand whether habitat availability affects PLD, we determined the PLD of 376 individuals from four reefs (Fitzroy, One Tree, Lamont and Heron, which are 4 to 20 km apart) over four years: 2008 before TC Hamish hit the reefs; coral cover was high and therefore suitable habitat was low; 2011 and 2012 after TC Hamish destroyed reefs and significantly increased rubble habitat was available; in 2015, when corals had partly regrown and less habitat was available than in 2011 and 2012 (Gerlach et al. 2021) (see Figure 3). The animals settled almost 3 days earlier in years when more habitat was available (Figure 3). PLD differed significantly between the different years (Kruskal‐Wallis test: *χ* ^2^ = 44.3, df = 3, *p* < 0.0001, for post hoc Dunn test see Table 3), but not among different reefs of the CBR reefs (One Tree, Heron, Fitzroy and Lamont) *χ* ^2^ = 4.77, df = 3, *p* = 0.19. PLD was highest in 2008 (PLD 23.3 ± 4.6 days) and 2015 (PLD mean = 21.9 ± 3.8 days) and lowest in 2011 (PLD mean = 20.5 ± 4.3 days) and 2012 (PLD mean = 20.4 ± 4.5 days) (Figure 3).

**TABLE 3:** Results of pairwise Dunn tests comparing the PLD of 4 different years.

	2008	2011	2012
2011	*p* < 0.001 *Z* = −6.27		
2012	*p* < 0.001 Z = −3.64	*p* = 1 Z = −0.17	
2015	*p* = 0.136 *Z* = −2.27	*p* = 0.021 *Z* = 2.91	*p* = 0.26 *Z* = 28

*Note:* *p*‐value is Bonferroni corrected and *Z* value is given. Test was carried out using rstatix (Kassambara 2023).

## 4 Discussion

### 4.1 PolyQ Variability in 20 Pomacentrid Species and Its Correlation With Pelagic Larval Duration (PLD)

To understand the potential influence of *clock* genes on PLD in coral reef fish, we characterised two closely linked glutamine repeat regions (PolyQ and Qrich) of the *clock* gene in 20 pomacentrid species. To avoid the influence of latitude on PLD, we focussed as much as possible on species originating from a relatively small geographical area of the Capricorn Bunker Reefs at the southern tip of the Great Barrier Reef, Australia. Species of this family of coral reef fish differed in their PLD in the range of 11 ( *Amphiprion melanopus* ) to 31.8 days ( *Dascyllus reticulatus* ). All species showed individual variation in the length of the PolyQ and Qrich domain. Using PGLS, we showed that PLD was significantly correlated with the length of PolyQ and Qrich (adjusted *R* ^2^ = 0.42); species with longer PolyQ had a longer PLD and the number of glutamine repeats in Qrich was negatively correlated with PLD, suggesting that both PolyQ and Qrich may be involved in the timing of developmental and migratory behaviour of Pomacentrids in general. However, note that the sample size of individuals per species (*n* = 1–6) is not sufficient to capture the entire genetic variability of the PolyQ region.

Kingsford et al. (2022) showed that size rather than age determined the settlement of juvenile fish. Factors such as water temperature, oceanography, and choice of settlement site were likely responsible for the 55%–60% variation in PLD. While in our phylogenetic approach considering 20 species of Pomacentridae, the interspecies correlation between PolyQ and PLD was significant, the unexplained variance (explained variance: adjusted *R* ^2^ = 0.42) was relatively large, and environmental factors might explain the remaining variance.

### 4.2 PLD Is Not Correlated With the Frequency of Specific PolyQ Alleles in *P. coelestis*

To understand whether variability in the length of PolyQ could explain individual differences in PLD within a species, we analysed the glutamine repeat‐PLD relationship in *P. coelestis* for PolyQ and Qrich. Interestingly, we found no correlation between the length of PolyQ or Qrich and PLD, which varied between 10 and 42 days. This might suggest that these polyglutamine repeats do not play an all‐important role in controlling the timing of development and settlement behaviour—at least not in *P. coelestis* .

During the sampling period (2009), tropical cyclone Hamish hit the Capricorn Bunker reefs and destroyed much of the coral, significantly increasing the suitable habitat for potential colonisation by *P. coelestis* . Fish settled significantly earlier (1.5–3 days on average) when more habitat was available and therefore easier to find. This is further evidence that return and settlement are not tied to a strict, genetic determination, but are influenced by a variety of ecological and behavioural factors—and can be delayed by days, as e.g., in the labrid coral reef fish *Thalassoma bifasciatum* which can delay metamorphosis for several weeks (Victor 1986). Our sampling of *P. coelestis* at the CBR took place in January in all years, so we conclude that water temperature and food availability should be roughly comparable between years, so that habitat availability could be the main factor for driving PLD. Our findings that *P. coelestis* colonises earlier when habitat availability is higher could therefore complement the results of the study by Kingsford et al. (2022). Rapid settlement could be even more important for the extremely short‐lived *P. coelestis* , which is displaced by other species as soon as coral cover increases (Gerlach et al. 2021).

There are only a limited number of studies on the potential impacts of PolyQ on fish migration, mostly focusing on salmon such as Chinook salmon ( *Oncorhynchus tshawytscha* ) (O'Malley and Banks 2008) or other Pacific salmon species (*Oncorhynchus* spp.) (O'Malley et al. 2010). While these studies provided evidence for an existing latitudinal cline, which is assumed to influence the timing of migration, direct evidence for a correlation is scarce. In Chinook salmon, PolyQ allele frequencies differed between spring and fall migratory runs (O'Malley et al. 2007), but Evans et al. (2015) found no association of PolyQ in early‐ and late‐arrivals in a reintroduced Chinook salmon population. The overall phenotype differs massively from that of the pomacentrids studied here, as salmon often remain in the sea for several years before returning to their spawning grounds.

### 4.3 Comparison of PolyQ/Qrich Sequence Properties of Fish and Birds

At least partially, the genetic structure of PolyQ/Qrich resembles the big picture of the phylogenetic relationship between the species, as e.g., *Abudefduf sexfasciatus* is the most distant species (McCord et al. 2021); it lacks the first region of PolyQ. The length and structure of PolyQ and Qrich in *Acanthochromis polyacanthus*, the only species we studied without a dispersal phase and thus with the greatest deviation from the usual migratory phenotype, did not differ from those of the other species.

Compared to pomacentrids, in birds, the PolyQ is considerably shorter. Bazzi et al. (2016) found only one allele in total consisting of 4 glutamine repeats in the European bee eater ( *Merops apiaster* ); The longest recorded glutamine repeat in birds contained 17 glutamines and was found in the blue tit (*Cyanister caeruleus*) (Johnsen et al. 2007), while in Pomacentridae we found a minimum of 17 glutamine repeats when considering only the region we called PolyQ. In the combination of PolyQ and Qrich, the smallest fragment contained 32 glutamine repeats and was thus almost twice as long as the longest known avian PolyQ fragment. PolyQ is not only longer, but also more variable in the Pomacentridae than in most bird species; it is even monomorphic in e.g., the great tit ( *Parus major* ) (Laine et al. 2019). In their review on birds, Le Clercq et al. (2023) provided evidence of a putative association between e.g., PolyQ Clock gene variations and autumn migration. Similar to our findings, they concluded that this candidate gene is not a diagnostic marker to distinguish migratory from sedentary species.

In pomacentrids, we found a large proportion of heterozygous animals (between 30% and 50% of Qrich respectively PolyQ in species comparison) and high allele variability in *P. coelestis* . This high variability in Pomacentrids compared to birds might enable a higher plasticity in migratory patterns in fish. In the barn swallow ( *Hirundo rustica* ), Bazzi et al. (2015) hypothesised that PolyQ and timing may be related to fitness and that these genes are under stronger selection pressure and are therefore less variable in some bird species than in others.

## 5 Conclusion

Our cross‐species analysis suggests that glutamine repeats associated with clock genes and developmental processes driven by them may indeed play a role in setting the overall time frame for colonisation, but their influence could be masked by factors such as habitat availability, food, oceanography, and ambient temperature. Knowledge of the flexibility of PLD in marine organisms in general would help to better assess their chances of survival under changing environmental conditions.

## Author Contributions

**Gregor Schalm:** conceptualization (equal), data curation (equal), formal analysis (equal), methodology (equal), writing – original draft (equal). **Simon Kaefer:** formal analysis (equal). **Philipp Krämer:** formal analysis (equal). **Anna‐Lena Jäger:** data curation (equal), methodology (equal). **Michael J. Kingsford:** data curation (equal), methodology (equal). **Gabriele Gerlach:** conceptualization (equal), funding acquisition (equal), methodology (equal), project administration (equal), writing – review and editing (equal).

## Conflicts of Interest

The authors declare no conflicts of interest.

## Supporting information


**Figure S1.** Transverse section of a *Pomacentrus coelestis* sagittal otolith (scale bar 50 μm). Black guideline highlights the counted section from primordium to settlement mark; white dots mark 19 daily rings and white arrow points at the settlement mark. Photograph was taken using a Sony 𝛼 6500 camera mounted on a transmitted‐light microscope (Leica DMLB).
**Figure S2.** Pelagic larval duration dependent on glutamine repeat numbers of PolyQ and Qrich of *Pomacentrus coelestis* . A and B: When alleles of PolyQ and Qrich per individual were of different fragment length we run the analysis for the shorter and the longer and the mean length of both. A and B are combined for all years and C and D are separated by year of sampling. PLD was determined by counting daily otolith rings (increments) from the primordium to the settlement mark. No significant correlation was found.
**Table S1.** PolyQ/Qrich alleles (PolyQ_A1/A2) and (Qrich‐A1/A2) of *Pomacentrus coelestis* from different reefs and years and individual Pelagic Larval Duration (PLD).
**Table S2.** PLD of *P. coelestis* from up to 4 reefs in 4 different years.

## Data Availability

Upon acceptance, gene sequence data will be made available on the NCBI Nucleotide database. Counting data and analysis script (as. Rmarkdown) will be made available as Supporting Information and in a publicly available cloud folder. The research described in the publication complies with all relevant national laws, implementing the Convention of Biological Diversity and Nagoya Protocol agreements.
