# Supplementary material for: PolyQ Length of the Clock Gene Is Correlated With Pelagic Larval Duration in the Damselfishes (Pomacentridae), but Within a Species Habitat Availability Counts
Source: Ecol Evol. 2025 Apr 25;15(4):e71259. doi: 10.1002/ece3.71259 (PMC12031889; doi:10.1002/ece3.71259)
Supplement: Supplementary file 1 — Figure S1. Transverse section of a Pomacentrus coelestis sagittal otolith (scale bar 50 μm). Black guideline highlights the counted section from primordium to settlement mark; white dots mark 19 daily rings and white arrow points at the settlement mark. Photograph was taken using a Sony 𝛼 6500 camera mounted on a transmitted‐light microscope (Leica DMLB). Figure S2. Pelagic larval duration dependent on glutamine repeat numbers of PolyQ and Qrich of Pomacentrus coelestis . A and B: When alleles of PolyQ and Qrich per individual were of different fragment length we run the analysis for the shorter and the longer and the mean length of both. A and B are combined for all years and C and D are separated by year of sampling. PLD was determined by counting daily otolith rings (increments) from the primordium to the settlement mark. No significant correlation was found. Table S1. PolyQ/Qrich alleles (PolyQ_A1/A2) and (Qrich‐A1/A2) of Pomacentrus coelestis from different reefs and years and individual Pelagic Larval Duration (PLD). Table S2. PLD of P. coelestis from up to 4 reefs in 4 different years. [file ECE3-15-e71259-s001.docx]

FIGURE S1: Transverse section of a Pomacentrus coelestis sagittal otolith (scale bar 50 µm). Black guideline highlights the counted section from primordium to settlement mark; white dots mark 19 daily rings and white arrow points at the settlement mark. Photograph was taken using a Sony $\alpha$6500 camera mounted on a transmitted-light microscope (Leica DMLB).


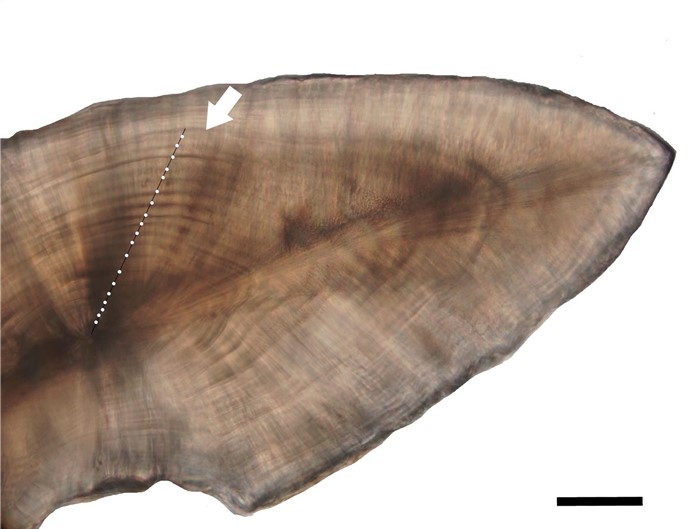


FIGURE S2: Pelagic larval duration dependent on glutamine repeat numbers of PolyQ and Qrich of Pomacentrus coelestis. A and B: When alleles of PolyQ and Qrich per individual were of different fragment length we run the analysis for the shorter and the longer and the mean length of both. A and B are combined for all years and C and D are separated by year of sampling. PLD was determined by counting daily otolith rings (increments) from the primordium to the settlement mark. No significant correlation was found.

| 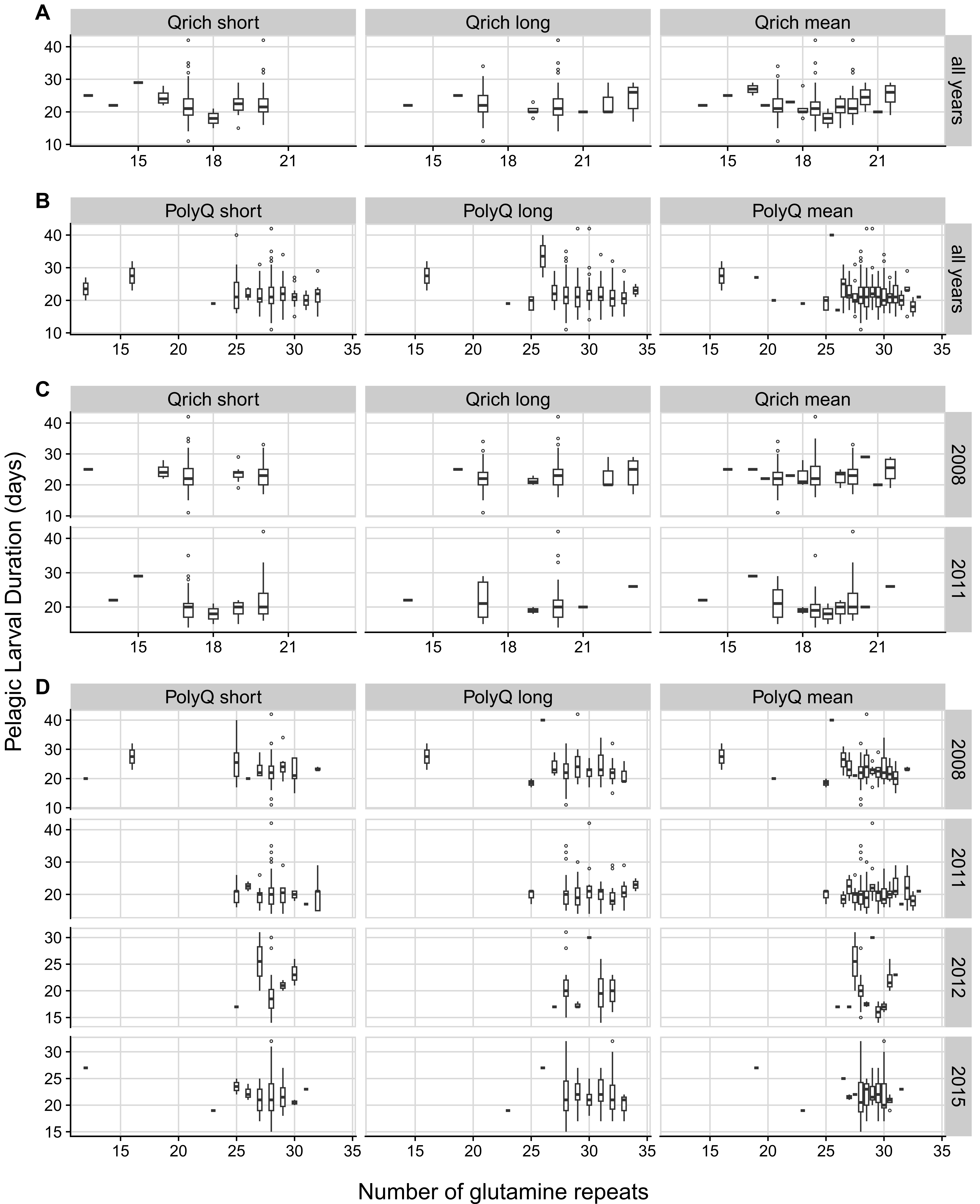 | . |
| --- | --- |

Table

Table S1

PolyQ/Qrich alleles (PolyQ_A1/A2) and Qrich-A1/A2) of *Pomacentrus coelestis* from different reefs and years and individual Pelagic Larval Duration (PLD).

| **Sample_ID** | **Year** | **Reef** | **PLD** | **PolyQ_A1** | **PolyQ_A2** | **Qrich_A1** | **Qrich_A2** |
| --- | --- | --- | --- | --- | --- | --- | --- |
| F.08.PC.09 | 2008 | Fitzroy | 28 | 240 | 243 | 277 | 289 |
| F.08.PC.11 | 2008 | Fitzroy | 26 | 231 | 243 | 289 | 289 |
| F.08.PC.13 | 2008 | Fitzroy | 20 | 240 | 240 | 289 | 289 |
| F.08.PC.17 | 2008 | Fitzroy | 23 | 252 | 252 | 277 | 286 |
| F.08.PC.19 | 2008 | Fitzroy | 23 | 240 | 240 | 289 | 289 |
| F.08.PC.20 | 2008 | Fitzroy | 28 | 240 | 240 | 289 | 298 |
| F.08.PC.21 | 2008 | Fitzroy | 24 | 252 | 252 | 280 | 280 |
| F.08.PC.22 | 2008 | Fitzroy | 21 | 240 | 240 | 280 | 280 |
| F.08.PC.23 | 2008 | Fitzroy | 22 | 240 | 240 | 289 | 289 |
| F.08.PC.25 | 2008 | Fitzroy | 23 | 240 | 246 | 286 | 289 |
| F.08.PC.27 | 2008 | Fitzroy | 22 | 243 | 243 | 277 | 280 |
| F.08.PC.28 | 2008 | Fitzroy | 22 | 240 | 240 | 280 | 289 |
| F.08.PC.33 | 2008 | Fitzroy | 23 | 240 | 240 | 289 | 298 |
| F.08.PC.38 | 2008 | Fitzroy | 22 | 240 | 240 | 280 | 280 |
| F.08.PC.39 | 2008 | Fitzroy | 23 | 240 | 252 | 280 | 280 |
| F.08.PC.42 | 2008 | Fitzroy | 24 | 240 | 240 | 289 | 289 |
| F.08.PC.43 | 2008 | Fitzroy | 24 | 240 | 243 | 280 | 280 |
| F.08.PC.45 | 2008 | Fitzroy | 28 | 240 | 243 | 280 | 289 |
| F.08.PC.46 | 2008 | Fitzroy | 20 | 240 | 243 | 289 | 295 |
| F.08.PC.47 | 2008 | Fitzroy | 23 | 240 | 243 | 280 | 289 |
| F.08.PC.50 | 2008 | Fitzroy | 23 | 240 | 246 | 289 | 289 |
| F1.11.PC.173 | 2011 | Fitzroy | 23 | 240 | 246 | 280 | 289 |
| F1.11.PC.175 | 2011 | Fitzroy | 26 | 237 | 240 | 289 | 289 |
| F1.11.PC.179 | 2011 | Fitzroy | 22 | 240 | 246 | 289 | 289 |
| F2.11.PC.209 | 2011 | Fitzroy | 27 | 240 | 243 | 280 | 280 |
| F2.11.PC.210 | 2011 | Fitzroy | 35 | 240 | 240 | 280 | 289 |
| F2.11.PC.218 | 2011 | Fitzroy | 20 | 240 | 240 | 289 | 292 |
| He1.08.PC.01 | 2008 | Heron | 20 | 246 | 246 | 280 | 289 |
| He1.08.PC.02 | 2008 | Heron | 27 | 240 | 240 | 280 | 289 |
| He1.08.PC.03 | 2008 | Heron | 25 | 240 | 240 | 289 | 289 |
| He1.08.PC.06 | 2008 | Heron | 29 | 237 | 237 | 280 | 289 |
| He1.08.PC.07 | 2008 | Heron | 29 | 240 | 249 | 286 | 295 |
| He1.08.PC.11 | 2008 | Heron | 24 | 243 | 246 | 280 | 280 |
| He1.08.PC.13 | 2008 | Heron | 23 | 237 | 237 | 280 | 280 |
| He1.08.PC.14 | 2008 | Heron | 27 | 246 | 246 | 289 | 289 |
| He1.08.PC.15 | 2008 | Heron | 26 | 240 | 240 | 280 | 289 |
| He1.08.PC.16 | 2008 | Heron | 21 | 240 | 240 | 286 | 289 |
| He1.08.PC.17 | 2008 | Heron | 26 | 240 | 240 | 280 | 280 |
| He1.11.PC.103 | 2011 | Heron | 15 | 240 | 240 | 289 | 289 |
| He1.11.PC.105 | 2011 | Heron | 22 | 240 | 240 | 289 | 289 |
| He1.11.PC.107 | 2011 | Heron | 14 | 240 | 245 | 280 | 289 |
| He1.11.PC.108 | 2011 | Heron | 24 | 234 | 240 | 289 | 292 |
| He1.11.PC.109 | 2011 | Heron | 18 | 243 | 245 | 280 | 289 |
| He1.11.PC.110 | 2011 | Heron | 24 | 240 | 240 | 289 | 289 |
| He1.11.PC.111 | 2011 | Heron | 18 | 243 | 249 | 289 | 289 |
| He1.11.PC.112 | 2011 | Heron | 17 | 249 | 252 | 280 | 280 |
| He1.11.PC.113 | 2011 | Heron | 24 | 240 | 249 | 289 | 289 |
| He1.11.PC.114 | 2011 | Heron | 33 | 240 | 240 | 289 | 289 |
| He1.11.PC.117 | 2011 | Heron | 19 | 240 | 249 | 280 | 289 |
| He1.11.PC.118 | 2011 | Heron | 20 | 237 | 240 | 289 | 289 |
| He1.11.PC.119 | 2011 | Heron | 14 | 240 | 249 | 280 | 289 |
| He2.08.PC.09 | 2008 | Heron | 21 | 240 | 252 | 289 | 289 |
| He2.08.PC.10 | 2008 | Heron | 19 | 240 | 252 | 280 | 289 |
| He2.08.PC.11 | 2008 | Heron | 27 | 240 | 243 | 280 | 289 |
| He2.08.PC.15 | 2008 | Heron | 20 | 240 | 252 | 280 | 289 |
| He2.08.PC.17 | 2008 | Heron | 24 | 240 | 252 | 286 | 289 |
| He2.08.PC.18 | 2008 | Heron | 24 | 240 | 240 | 286 | 289 |
| He2.08.PC.20 | 2008 | Heron | 24 | 240 | 240 | 289 | 289 |
| He2.08.PC.21 | 2008 | Heron | 28 | 231 | 240 | 289 | 289 |
| He2.08.PC.25 | 2008 | Heron | 28 | 240 | 243 | 280 | 289 |
| He2.08.PC.28 | 2008 | Heron | 23 | 240 | 240 | 289 | 289 |
| He2.08.PC.31 | 2008 | Heron | 17 | 240 | 240 | 289 | 289 |
| He2.08.PC.33 | 2008 | Heron | 19 | 240 | 255 | 280 | 289 |
| He2.08.PC.34 | 2008 | Heron | 17 | 240 | 249 | 280 | 289 |
| He2.08.PC.36 | 2008 | Heron | 22 | 240 | 243 | 280 | 289 |
| He2.08.PC.37 | 2008 | Heron | 18 | 240 | 252 | 280 | 289 |
| He2.11.PC.132 | 2011 | Heron | 21 | 240 | 245 | 283 | 289 |
| He2.11.PC.133 | 2011 | Heron | 20 | 243 | 245 | 280 | 286 |
| He2.11.PC.134 | 2011 | Heron | 26 | 240 | 240 | 289 | 298 |
| He2.11.PC.139 | 2011 | Heron | 17 | 240 | 243 | 280 | 289 |
| He2.11.PC.141 | 2011 | Heron | 26 | 231 | 243 | 280 | 289 |
| He2.11.PC.143 | 2011 | Heron | 22 | 240 | 243 | 283 | 292 |
| He2.11.PC.144 | 2011 | Heron | 22 | 240 | 246 | 280 | 289 |
| He2.11.PC.145 | 2011 | Heron | 22 | 243 | 249 | 289 | 289 |
| He2.11.PC.147 | 2011 | Heron | 18 | 237 | 240 | 289 | 289 |
| He2.11.PC.148 | 2011 | Heron | 20 | 240 | 240 | 289 | 289 |
| He2.11.PC.149 | 2011 | Heron | 24 | 240 | 249 | 289 | 289 |
| He4.11.PC.154 | 2011 | Heron | 14 | 240 | 243 | 280 | 289 |
| He4.11.PC.155 | 2011 | Heron | 15 | 240 | 243 | 283 | 289 |
| He4.11.PC.156 | 2011 | Heron | 24 | 240 | 240 | 289 | 289 |
| He4.11.PC.157 | 2011 | Heron | 16 | 240 | 243 | 289 | 289 |
| He4.11.PC.158 | 2011 | Heron | 20 | 240 | 240 | 280 | 289 |
| He4.11.PC.159 | 2011 | Heron | 19 | 240 | 243 | 280 | 289 |
| L1.08.PC.01 | 2008 | Lamont | 23 | 243 | 252 | 280 | 289 |
| L1.08.PC.03 | 2008 | Lamont | 34 | 243 | 249 | 280 | 280 |
| L1.08.PC.08 | 2008 | Lamont | 19 | 243 | 255 | 280 | 280 |
| L1.08.PC.13 | 2008 | Lamont | 30 | 240 | 243 | 280 | 280 |
| L1.08.PC.15 | 2008 | Lamont | 20 | 193 | 243 | 280 | 280 |
| L1.11.PC.224 | 2011 | Lamont | 20 | 240 | 243 | 280 | 289 |
| L1.11.PC.225 | 2011 | Lamont | 18 | 240 | 252 | 289 | 289 |
| L1.11.PC.226 | 2011 | Lamont | 20 | 240 | 246 | 289 | 289 |
| L1.11.PC.228 | 2011 | Lamont | 21 | 240 | 243 | 289 | 289 |
| L1.11.PC.229 | 2011 | Lamont | 20 | 240 | 243 | 280 | 289 |
| L1.11.PC.231 | 2011 | Lamont | 21 | 231 | 231 | 280 | 289 |
| L1.11.PC.232 | 2011 | Lamont | 16 | 240 | 243 | 280 | 289 |
| L1.11.PC.233 | 2011 | Lamont | 16 | 240 | 240 | 289 | 289 |
| L1.11.PC.234 | 2011 | Lamont | 21 | 252 | 255 | 280 | 280 |
| L1.11.PC.235 | 2011 | Lamont | 20 | 240 | 240 | 280 | 289 |
| L2.08.PC.12 | 2008 | Lamont | 42 | 240 | 243 | 280 | 289 |
| L2.08.PC.13 | 2008 | Lamont | 29 | 240 | 243 | 289 | 298 |
| L2.08.PC.14 | 2008 | Lamont | 15 | 246 | 252 | 280 | 280 |
| L2.08.PC.16 | 2008 | Lamont | 21 | 231 | 240 | 280 | 289 |
| L2.08.PC.17 | 2008 | Lamont | 21 | 240 | 240 | 289 | 289 |
| L2.08.PC.18 | 2008 | Lamont | 20 | 240 | 240 | 280 | 289 |
| L2.08.PC.19 | 2008 | Lamont | 21 | 240 | 243 | 280 | 280 |
| L2.08.PC.20 | 2008 | Lamont | 20 | 240 | 249 | 280 | 289 |
| L2.08.PC.21 | 2008 | Lamont | 23 | 205 | 205 | 289 | 289 |
| L2.08.PC.22 | 2008 | Lamont | 22 | 240 | 249 | 280 | 289 |
| L2.08.PC.23 | 2008 | Lamont | 32 | 240 | 240 | 280 | 289 |
| L2.08.PC.24 | 2008 | Lamont | 19 | 240 | 240 | 289 | 289 |
| L2.08.PC.28 | 2008 | Lamont | 21 | 246 | 252 | 280 | 280 |
| L2.08.PC.29 | 2008 | Lamont | 20 | 240 | 243 | 280 | 289 |
| L2.08.PC.31 | 2008 | Lamont | 22 | 240 | 240 | 280 | 289 |
| L2.08.PC.32 | 2008 | Lamont | 19 | 240 | 240 | 289 | 298 |
| L2.08.PC.33 | 2008 | Lamont | 21 | 240 | 240 | 289 | 289 |
| L2.08.PC.34 | 2008 | Lamont | 19 | 240 | 240 | 289 | 289 |
| L2.08.PC.42 | 2008 | Lamont | 18 | 240 | 240 | 289 | 289 |
| L2.08.PC.43 | 2008 | Lamont | 16 | 240 | 240 | 280 | 289 |
| L2.08.PC.44 | 2008 | Lamont | 21 | 240 | 252 | 280 | 289 |
| L2.08.PC.45 | 2008 | Lamont | 20 | 243 | 252 | 280 | 280 |
| L2.11.PC.240 | 2011 | Lamont | 20 | 240 | 240 | 289 | 289 |
| L2.11.PC.242 | 2011 | Lamont | 21 | 234 | 240 | 280 | 289 |
| L2.11.PC.243 | 2011 | Lamont | 18 | 240 | 243 | 289 | 289 |
| L2.11.PC.244 | 2011 | Lamont | 20 | 237 | 240 | 289 | 289 |
| L2.11.PC.246 | 2011 | Lamont | 17 | 240 | 243 | 280 | 289 |
| L2.11.PC.248 | 2011 | Lamont | 18 | 240 | 246 | 289 | 289 |
| L2.11.PC.250 | 2011 | Lamont | 20 | 240 | 240 | 280 | 280 |
| L2.11.PC.251 | 2011 | Lamont | 17 | 240 | 240 | 280 | 280 |
| L2.11.PC.253 | 2011 | Lamont | 19 | 240 | 240 | 289 | 289 |
| L2.11.PC.254 | 2011 | Lamont | 18 | 240 | 252 | 280 | 286 |
| L3.11.PC.256 | 2011 | Lamont | 28 | 240 | 252 | 280 | 280 |
| L3.11.PC.257 | 2011 | Lamont | 21 | 240 | 255 | 280 | 280 |
| L3.11.PC.258 | 2011 | Lamont | 20 | 237 | 240 | 289 | 289 |
| L3.11.PC.259 | 2011 | Lamont | 19 | 240 | 243 | 280 | 289 |
| L3.11.PC.260 | 2011 | Lamont | 21 | 240 | 249 | 286 | 289 |
| L3.11.PC.261 | 2011 | Lamont | 15 | 252 | 255 | 280 | 280 |
| L3.11.PC.263 | 2011 | Lamont | 18 | 240 | 240 | 289 | 289 |
| L3.11.PC.267 | 2011 | Lamont | 20 | 240 | 240 | 289 | 289 |
| L3.11.PC.268 | 2011 | Lamont | 17 | 240 | 240 | 289 | 289 |
| OTI2.11.PC.286 | 2011 | OTI | 17 | 240 | 252 | 280 | 289 |
| OTI2.11.PC.290 | 2011 | OTI | 42 | 240 | 246 | 289 | 289 |
| OTI2.11.PC.291 | 2011 | OTI | 16 | 240 | 240 | 280 | 289 |
| OTI2.11.PC.293 | 2011 | OTI | 16 | 243 | 252 | 280 | 280 |
| OTI2.11.PC.296 | 2011 | OTI | 17 | 240 | 252 | 280 | 289 |
| OTI2.11.PC.297 | 2011 | OTI | 16 | 240 | 243 | 289 | 289 |
| OTI2.11.PC.300 | 2011 | OTI | 20 | 240 | 252 | 280 | 289 |
| OTI2.11.PC.302 | 2011 | OTI | 28 | 240 | 246 | 289 | 289 |
| OTI2.11.PC.304 | 2011 | OTI | 29 | 252 | 252 | 274 | 280 |
| OTI2.11.PC.305 | 2011 | OTI | 23 | 240 | 255 | 280 | 280 |
| OTI2.11.PC.309 | 2011 | OTI | 22 | 240 | 243 | 289 | 289 |
| OTI2.11.PC.310 | 2011 | OTI | 29 | 243 | 255 | 280 | 280 |
| OTI2.11.PC.311 | 2011 | OTI | 23 | 240 | 240 | 289 | 289 |
| OTI2.11.PC.313 | 2011 | OTI | 22 | 243 | 243 | 271 | 271 |
| OTINE.11.PC.320 | 2011 | OTI | 19 | 240 | 240 | 286 | 289 |
| OTINE.11.PC.329 | 2011 | OTI | 16 | 240 | 240 | 289 | 289 |
| OTINE.11.PC.330 | 2011 | OTI | 17 | 240 | 243 | 280 | 289 |

Table S2 PLD of *P. coelestis* from up to 4 reefs in 4 different years

| **Year** | **Reef** | **Sample_ID** | **Population** | **PLD** |
| --- | --- | --- | --- | --- |
| 2008 | Fitzroy | F.08.PC.06 | Fitzroy08 | 17 |
| 2008 | Fitzroy | F.08.PC.10 | Fitzroy08 | 18 |
| 2008 | Fitzroy | F.08.PC.30 | Fitzroy08 | 19 |
| 2008 | Fitzroy | F.08.PC.13 | Fitzroy08 | 20 |
| 2008 | Fitzroy | F.08.PC.35 | Fitzroy08 | 20 |
| 2008 | Fitzroy | F.08.PC.46 | Fitzroy08 | 20 |
| 2008 | Fitzroy | F.08.PC.14 | Fitzroy08 | 21 |
| 2008 | Fitzroy | F.08.PC.22 | Fitzroy08 | 21 |
| 2008 | Fitzroy | F.08.PC.29 | Fitzroy08 | 21 |
| 2008 | Fitzroy | F.08.PC.07 | Fitzroy08 | 22 |
| 2008 | Fitzroy | F.08.PC.23 | Fitzroy08 | 22 |
| 2008 | Fitzroy | F.08.PC.27 | Fitzroy08 | 22 |
| 2008 | Fitzroy | F.08.PC.28 | Fitzroy08 | 22 |
| 2008 | Fitzroy | F.08.PC.32 | Fitzroy08 | 22 |
| 2008 | Fitzroy | F.08.PC.38 | Fitzroy08 | 22 |
| 2008 | Fitzroy | F.08.PC.17 | Fitzroy08 | 23 |
| 2008 | Fitzroy | F.08.PC.19 | Fitzroy08 | 23 |
| 2008 | Fitzroy | F.08.PC.25 | Fitzroy08 | 23 |
| 2008 | Fitzroy | F.08.PC.33 | Fitzroy08 | 23 |
| 2008 | Fitzroy | F.08.PC.39 | Fitzroy08 | 23 |
| 2008 | Fitzroy | F.08.PC.47 | Fitzroy08 | 23 |
| 2008 | Fitzroy | F.08.PC.50 | Fitzroy08 | 23 |
| 2008 | Fitzroy | F.08.PC.04 | Fitzroy08 | 24 |
| 2008 | Fitzroy | F.08.PC.08 | Fitzroy08 | 24 |
| 2008 | Fitzroy | F.08.PC.15 | Fitzroy08 | 24 |
| 2008 | Fitzroy | F.08.PC.21 | Fitzroy08 | 24 |
| 2008 | Fitzroy | F.08.PC.34 | Fitzroy08 | 24 |
| 2008 | Fitzroy | F.08.PC.42 | Fitzroy08 | 24 |
| 2008 | Fitzroy | F.08.PC.43 | Fitzroy08 | 24 |
| 2008 | Fitzroy | F.08.PC.16 | Fitzroy08 | 25 |
| 2008 | Fitzroy | F.08.PC.24 | Fitzroy08 | 25 |
| 2008 | Fitzroy | F.08.PC.11 | Fitzroy08 | 26 |
| 2008 | Fitzroy | F.08.PC.02 | Fitzroy08 | 27 |
| 2008 | Fitzroy | F.08.PC.05 | Fitzroy08 | 27 |
| 2008 | Fitzroy | F.08.PC.18 | Fitzroy08 | 27 |
| 2008 | Fitzroy | F.08.PC.09 | Fitzroy08 | 28 |
| 2008 | Fitzroy | F.08.PC.20 | Fitzroy08 | 28 |
| 2008 | Fitzroy | F.08.PC.45 | Fitzroy08 | 28 |
| 2008 | Fitzroy | F.08.PC.01 | Fitzroy08 | 31 |
| 2008 | Fitzroy | F.08.PC.03 | Fitzroy08 | 38 |
| 2008 | Heron | He2.08.PC.31 | Heron08 | 17 |
| 2008 | Heron | He2.08.PC.34 | Heron08 | 17 |
| 2008 | Heron | He2.08.PC.37 | Heron08 | 18 |
| 2008 | Heron | He2.08.PC.10 | Heron08 | 19 |
| 2008 | Heron | He2.08.PC.33 | Heron08 | 19 |
| 2008 | Heron | He1.08.PC.01 | Heron08 | 20 |
| 2008 | Heron | He1.08.PC.18 | Heron08 | 20 |
| 2008 | Heron | He2.08.PC.04 | Heron08 | 20 |
| 2008 | Heron | He2.08.PC.14 | Heron08 | 20 |
| 2008 | Heron | He2.08.PC.15 | Heron08 | 20 |
| 2008 | Heron | He1.08.PC.16 | Heron08 | 21 |
| 2008 | Heron | He2.08.PC.09 | Heron08 | 21 |
| 2008 | Heron | He2.08.PC.29 | Heron08 | 21 |
| 2008 | Heron | He1.08.PC.09 | Heron08 | 22 |
| 2008 | Heron | He2.08.PC.36 | Heron08 | 22 |
| 2008 | Heron | He1.08.PC.05 | Heron08 | 23 |
| 2008 | Heron | He1.08.PC.13 | Heron08 | 23 |
| 2008 | Heron | He2.08.PC.03 | Heron08 | 23 |
| 2008 | Heron | He2.08.PC.08 | Heron08 | 23 |
| 2008 | Heron | He2.08.PC.13 | Heron08 | 23 |
| 2008 | Heron | He2.08.PC.26 | Heron08 | 23 |
| 2008 | Heron | He2.08.PC.28 | Heron08 | 23 |
| 2008 | Heron | He1.08.PC.10 | Heron08 | 24 |
| 2008 | Heron | He1.08.PC.11 | Heron08 | 24 |
| 2008 | Heron | He1.08.PC.12 | Heron08 | 24 |
| 2008 | Heron | He2.08.PC.17 | Heron08 | 24 |
| 2008 | Heron | He2.08.PC.18 | Heron08 | 24 |
| 2008 | Heron | He2.08.PC.20 | Heron08 | 24 |
| 2008 | Heron | He1.08.PC.03 | Heron08 | 25 |
| 2008 | Heron | He1.08.PC.15 | Heron08 | 26 |
| 2008 | Heron | He1.08.PC.17 | Heron08 | 26 |
| 2008 | Heron | He1.08.PC.02 | Heron08 | 27 |
| 2008 | Heron | He1.08.PC.14 | Heron08 | 27 |
| 2008 | Heron | He2.08.PC.11 | Heron08 | 27 |
| 2008 | Heron | He1.08.PC.08 | Heron08 | 28 |
| 2008 | Heron | He2.08.PC.21 | Heron08 | 28 |
| 2008 | Heron | He2.08.PC.25 | Heron08 | 28 |
| 2008 | Heron | He1.08.PC.06 | Heron08 | 29 |
| 2008 | Heron | He1.08.PC.07 | Heron08 | 29 |
| 2008 | Heron | He2.08.PC.01 | Heron08 | 29 |
| 2008 | Heron | He2.08.PC.16 | Heron08 | 33 |
| 2008 | Heron | He1.08.PC.04 | Heron08 | 35 |
| 2008 | Heron | He2.08.PC.06 | Heron08 | 40 |
| 2008 | Lamont | L2.08.PC.40 | Lamont08 | 13 |
| 2008 | Lamont | L2.08.PC.14 | Lamont08 | 15 |
| 2008 | Lamont | L2.08.PC.43 | Lamont08 | 16 |
| 2008 | Lamont | L2.08.PC.42 | Lamont08 | 18 |
| 2008 | Lamont | L1.08.PC.08 | Lamont08 | 19 |
| 2008 | Lamont | L2.08.PC.24 | Lamont08 | 19 |
| 2008 | Lamont | L2.08.PC.32 | Lamont08 | 19 |
| 2008 | Lamont | L2.08.PC.34 | Lamont08 | 19 |
| 2008 | Lamont | L1.08.PC.15 | Lamont08 | 20 |
| 2008 | Lamont | L2.08.PC.18 | Lamont08 | 20 |
| 2008 | Lamont | L2.08.PC.20 | Lamont08 | 20 |
| 2008 | Lamont | L2.08.PC.29 | Lamont08 | 20 |
| 2008 | Lamont | L2.08.PC.45 | Lamont08 | 20 |
| 2008 | Lamont | L2.08.PC.15 | Lamont08 | 21 |
| 2008 | Lamont | L2.08.PC.16 | Lamont08 | 21 |
| 2008 | Lamont | L2.08.PC.17 | Lamont08 | 21 |
| 2008 | Lamont | L2.08.PC.19 | Lamont08 | 21 |
| 2008 | Lamont | L2.08.PC.28 | Lamont08 | 21 |
| 2008 | Lamont | L2.08.PC.33 | Lamont08 | 21 |
| 2008 | Lamont | L2.08.PC.44 | Lamont08 | 21 |
| 2008 | Lamont | L1.08.PC.05 | Lamont08 | 22 |
| 2008 | Lamont | L2.08.PC.22 | Lamont08 | 22 |
| 2008 | Lamont | L2.08.PC.31 | Lamont08 | 22 |
| 2008 | Lamont | L1.08.PC.01 | Lamont08 | 23 |
| 2008 | Lamont | L2.08.PC.21 | Lamont08 | 23 |
| 2008 | Lamont | L2.08.PC.36 | Lamont08 | 23 |
| 2008 | Lamont | L1.08.PC.06 | Lamont08 | 24 |
| 2008 | Lamont | L2.08.PC.30 | Lamont08 | 24 |
| 2008 | Lamont | L1.08.PC.07 | Lamont08 | 28 |
| 2008 | Lamont | L2.08.PC.13 | Lamont08 | 29 |
| 2008 | Lamont | L1.08.PC.13 | Lamont08 | 30 |
| 2008 | Lamont | L1.08.PC.02 | Lamont08 | 31 |
| 2008 | Lamont | L2.08.PC.23 | Lamont08 | 32 |
| 2008 | Lamont | L1.08.PC.03 | Lamont08 | 34 |
| 2008 | Lamont | L2.08.PC.12 | Lamont08 | 42 |
| 2008 | OTI | EU.08.OTI.PC.07 | OTI08 | 11 |
| 2008 | OTI | EU.08.OTI.PC.12 | OTI08 | 16 |
| 2008 | OTI | EU.08.OTI2.PC.19 | OTI08 | 17 |
| 2008 | OTI | EU.08.OTI2.PC.33 | OTI08 | 17 |
| 2008 | OTI | EU.08.OTI.PC.06 | OTI08 | 18 |
| 2008 | OTI | EU.08.OTI2.PC.37 | OTI08 | 19 |
| 2008 | OTI | EU.08.OTI.PC.04 | OTI08 | 20 |
| 2008 | OTI | EU.08.OTI.PC.11 | OTI08 | 20 |
| 2008 | OTI | EU.08.OTI2.PC.12 | OTI08 | 20 |
| 2008 | OTI | EU.08.OTI2.PC.21 | OTI08 | 20 |
| 2008 | OTI | EU.08.OTI2.PC.25 | OTI08 | 20 |
| 2008 | OTI | EU.08.OTI2.PC.28 | OTI08 | 20 |
| 2008 | OTI | EU.08.OTI.PC.09 | OTI08 | 21 |
| 2008 | OTI | EU.08.OTI.PC.10 | OTI08 | 21 |
| 2008 | OTI | EU.08.OTI2.PC.04 | OTI08 | 21 |
| 2008 | OTI | EU.08.OTI2.PC.11 | OTI08 | 21 |
| 2008 | OTI | EU.08.OTI2.PC.13 | OTI08 | 21 |
| 2008 | OTI | EU.08.OTI2.PC.15 | OTI08 | 21 |
| 2008 | OTI | EU.08.OTI2.PC.17 | OTI08 | 21 |
| 2008 | OTI | EU.08.OTI.PC.03 | OTI08 | 22 |
| 2008 | OTI | EU.08.OTI2.PC.06 | OTI08 | 22 |
| 2008 | OTI | EU.08.OTI2.PC.08 | OTI08 | 23 |
| 2008 | OTI | EU.08.OTI2.PC.16 | OTI08 | 23 |
| 2008 | OTI | EU.08.OTI2.PC.18 | OTI08 | 23 |
| 2008 | OTI | EU.08.OTI2.PC.34 | OTI08 | 23 |
| 2008 | OTI | EU.08.OTI2.PC.35 | OTI08 | 23 |
| 2008 | OTI | EU.08.OTI2.PC.27 | OTI08 | 24 |
| 2008 | OTI | EU.08.OTI2.PC.23 | OTI08 | 25 |
| 2008 | OTI | EU.08.OTI2.PC.30 | OTI08 | 25 |
| 2008 | OTI | EU.08.OTI2.PC.39 | OTI08 | 25 |
| 2008 | OTI | EU.08.OTI2.PC.40 | OTI08 | 25 |
| 2008 | OTI | EU.08.OTI.PC.01 | OTI08 | 26 |
| 2008 | OTI | EU.08.OTI2.PC.14 | OTI08 | 26 |
| 2008 | OTI | EU.08.OTI2.PC.24 | OTI08 | 26 |
| 2008 | OTI | EU.08.OTI2.PC.31 | OTI08 | 26 |
| 2008 | OTI | EU.08.OTI.PC.05 | OTI08 | 27 |
| 2008 | OTI | EU.08.OTI2.PC.07 | OTI08 | 27 |
| 2008 | OTI | EU.08.OTI2.PC.10 | OTI08 | 27 |
| 2008 | OTI | EU.08.OTI2.PC.22 | OTI08 | 28 |
| 2008 | OTI | EU.08.OTI.PC.02 | OTI08 | 32 |
| 2008 | OTI | EU.08.OTI2.PC.03 | OTI08 | 32 |
| 2011 | Fitzroy | F1.11.PC.180 | Fitzroy11 | 15 |
| 2011 | Fitzroy | F1.11.PC.176 | Fitzroy11 | 16 |
| 2011 | Fitzroy | F1.11.PC.177 | Fitzroy11 | 16 |
| 2011 | Fitzroy | F1.11.PC.196 | Fitzroy11 | 16 |
| 2011 | Fitzroy | F1.11.PC.170 | Fitzroy11 | 17 |
| 2011 | Fitzroy | F1.11.PC.172 | Fitzroy11 | 17 |
| 2011 | Fitzroy | F1.11.PC.165 | Fitzroy11 | 18 |
| 2011 | Fitzroy | F1.11.PC.174 | Fitzroy11 | 18 |
| 2011 | Fitzroy | F1.11.PC.178 | Fitzroy11 | 19 |
| 2011 | Fitzroy | F2.11.PC.213 | Fitzroy11 | 19 |
| 2011 | Fitzroy | F2.11.PC.216 | Fitzroy11 | 19 |
| 2011 | Fitzroy | F1.11.PC.195 | Fitzroy11 | 20 |
| 2011 | Fitzroy | F2.11.PC.218 | Fitzroy11 | 20 |
| 2011 | Fitzroy | F1.11.PC.166 | Fitzroy11 | 21 |
| 2011 | Fitzroy | F1.11.PC.171 | Fitzroy11 | 21 |
| 2011 | Fitzroy | F1.11.PC.181 | Fitzroy11 | 21 |
| 2011 | Fitzroy | F2.11.PC.214 | Fitzroy11 | 21 |
| 2011 | Fitzroy | F2.11.PC.215 | Fitzroy11 | 21 |
| 2011 | Fitzroy | F2.11.PC.217 | Fitzroy11 | 21 |
| 2011 | Fitzroy | F1.11.PC.167 | Fitzroy11 | 22 |
| 2011 | Fitzroy | F1.11.PC.179 | Fitzroy11 | 22 |
| 2011 | Fitzroy | F1.11.PC.173 | Fitzroy11 | 23 |
| 2011 | Fitzroy | F1.11.PC.175 | Fitzroy11 | 26 |
| 2011 | Fitzroy | F2.11.PC.209 | Fitzroy11 | 27 |
| 2011 | Fitzroy | F1.11.PC.168 | Fitzroy11 | 30 |
| 2011 | Fitzroy | F2.11.PC.210 | Fitzroy11 | 35 |
| 2011 | Heron | He1.11.PC.107 | Heron11 | 14 |
| 2011 | Heron | He1.11.PC.119 | Heron11 | 14 |
| 2011 | Heron | He4.11.PC.154 | Heron11 | 14 |
| 2011 | Heron | He1.11.PC.103 | Heron11 | 15 |
| 2011 | Heron | He4.11.PC.155 | Heron11 | 15 |
| 2011 | Heron | He4.11.PC.157 | Heron11 | 16 |
| 2011 | Heron | He1.11.PC.112 | Heron11 | 17 |
| 2011 | Heron | He2.11.PC.139 | Heron11 | 17 |
| 2011 | Heron | He1.11.PC.109 | Heron11 | 18 |
| 2011 | Heron | He1.11.PC.111 | Heron11 | 18 |
| 2011 | Heron | He2.11.PC.147 | Heron11 | 18 |
| 2011 | Heron | He1.11.PC.117 | Heron11 | 19 |
| 2011 | Heron | He4.11.PC.159 | Heron11 | 19 |
| 2011 | Heron | He1.11.PC.118 | Heron11 | 20 |
| 2011 | Heron | He2.11.PC.133 | Heron11 | 20 |
| 2011 | Heron | He2.11.PC.148 | Heron11 | 20 |
| 2011 | Heron | He4.11.PC.158 | Heron11 | 20 |
| 2011 | Heron | He1.11.PC.101 | Heron11 | 21 |
| 2011 | Heron | He2.11.PC.132 | Heron11 | 21 |
| 2011 | Heron | He2.11.PC.142 | Heron11 | 21 |
| 2011 | Heron | He1.11.PC.104 | Heron11 | 22 |
| 2011 | Heron | He1.11.PC.105 | Heron11 | 22 |
| 2011 | Heron | He2.11.PC.143 | Heron11 | 22 |
| 2011 | Heron | He2.11.PC.144 | Heron11 | 22 |
| 2011 | Heron | He2.11.PC.145 | Heron11 | 22 |
| 2011 | Heron | He1.11.PC.108 | Heron11 | 24 |
| 2011 | Heron | He1.11.PC.110 | Heron11 | 24 |
| 2011 | Heron | He1.11.PC.113 | Heron11 | 24 |
| 2011 | Heron | He2.11.PC.149 | Heron11 | 24 |
| 2011 | Heron | He4.11.PC.156 | Heron11 | 24 |
| 2011 | Heron | He2.11.PC.138 | Heron11 | 25 |
| 2011 | Heron | He2.11.PC.134 | Heron11 | 26 |
| 2011 | Heron | He2.11.PC.141 | Heron11 | 26 |
| 2011 | Heron | He2.11.PC.131 | Heron11 | 31 |
| 2011 | Heron | He1.11.PC.114 | Heron11 | 33 |
| 2011 | Lamont | L3.11.PC.261 | Lamont11 | 15 |
| 2011 | Lamont | L1.11.PC.232 | Lamont11 | 16 |
| 2011 | Lamont | L1.11.PC.233 | Lamont11 | 16 |
| 2011 | Lamont | L3.11.PC.262 | Lamont11 | 16 |
| 2011 | Lamont | L2.11.PC.246 | Lamont11 | 17 |
| 2011 | Lamont | L2.11.PC.251 | Lamont11 | 17 |
| 2011 | Lamont | L3.11.PC.264 | Lamont11 | 17 |
| 2011 | Lamont | L3.11.PC.268 | Lamont11 | 17 |
| 2011 | Lamont | L1.11.PC.225 | Lamont11 | 18 |
| 2011 | Lamont | L2.11.PC.243 | Lamont11 | 18 |
| 2011 | Lamont | L2.11.PC.248 | Lamont11 | 18 |
| 2011 | Lamont | L2.11.PC.254 | Lamont11 | 18 |
| 2011 | Lamont | L3.11.PC.263 | Lamont11 | 18 |
| 2011 | Lamont | L2.11.PC.253 | Lamont11 | 19 |
| 2011 | Lamont | L3.11.PC.259 | Lamont11 | 19 |
| 2011 | Lamont | L1.11.PC.224 | Lamont11 | 20 |
| 2011 | Lamont | L1.11.PC.226 | Lamont11 | 20 |
| 2011 | Lamont | L1.11.PC.229 | Lamont11 | 20 |
| 2011 | Lamont | L1.11.PC.235 | Lamont11 | 20 |
| 2011 | Lamont | L2.11.PC.240 | Lamont11 | 20 |
| 2011 | Lamont | L2.11.PC.244 | Lamont11 | 20 |
| 2011 | Lamont | L2.11.PC.250 | Lamont11 | 20 |
| 2011 | Lamont | L3.11.PC.258 | Lamont11 | 20 |
| 2011 | Lamont | L3.11.PC.267 | Lamont11 | 20 |
| 2011 | Lamont | L1.11.PC.228 | Lamont11 | 21 |
| 2011 | Lamont | L1.11.PC.231 | Lamont11 | 21 |
| 2011 | Lamont | L1.11.PC.234 | Lamont11 | 21 |
| 2011 | Lamont | L2.11.PC.242 | Lamont11 | 21 |
| 2011 | Lamont | L3.11.PC.257 | Lamont11 | 21 |
| 2011 | Lamont | L3.11.PC.260 | Lamont11 | 21 |
| 2011 | Lamont | L1.11.PC.223 | Lamont11 | 24 |
| 2011 | Lamont | L3.11.PC.256 | Lamont11 | 28 |
| 2011 | OTI | OTINE.11.PC.326 | OTI11 | 15 |
| 2011 | OTI | OTI2.11.PC.291 | OTI11 | 16 |
| 2011 | OTI | OTI2.11.PC.293 | OTI11 | 16 |
| 2011 | OTI | OTI2.11.PC.297 | OTI11 | 16 |
| 2011 | OTI | OTILT.11.PC.338 | OTI11 | 16 |
| 2011 | OTI | OTINE.11.PC.329 | OTI11 | 16 |
| 2011 | OTI | OTI2.11.PC.286 | OTI11 | 17 |
| 2011 | OTI | OTI2.11.PC.296 | OTI11 | 17 |
| 2011 | OTI | OTINE.11.PC.323 | OTI11 | 17 |
| 2011 | OTI | OTINE.11.PC.330 | OTI11 | 17 |
| 2011 | OTI | OTINE.11.PC.331 | OTI11 | 17 |
| 2011 | OTI | OTINE.11.PC.315 | OTI11 | 19 |
| 2011 | OTI | OTINE.11.PC.320 | OTI11 | 19 |
| 2011 | OTI | OTINE.11.PC.328 | OTI11 | 19 |
| 2011 | OTI | OTI2.11.PC.295 | OTI11 | 20 |
| 2011 | OTI | OTI2.11.PC.300 | OTI11 | 20 |
| 2011 | OTI | OTINE.11.PC.318 | OTI11 | 20 |
| 2011 | OTI | OTINE.11.PC.319 | OTI11 | 20 |
| 2011 | OTI | OTINE.11.PC.321 | OTI11 | 20 |
| 2011 | OTI | OTILT.11.PC.335 | OTI11 | 21 |
| 2011 | OTI | OTINE.11.PC.316 | OTI11 | 21 |
| 2011 | OTI | OTINE.11.PC.317 | OTI11 | 21 |
| 2011 | OTI | OTINE.11.PC.333 | OTI11 | 21 |
| 2011 | OTI | OTI2.11.PC.309 | OTI11 | 22 |
| 2011 | OTI | OTI2.11.PC.313 | OTI11 | 22 |
| 2011 | OTI | OTINE.11.PC.324 | OTI11 | 22 |
| 2011 | OTI | OTI2.11.PC.305 | OTI11 | 23 |
| 2011 | OTI | OTI2.11.PC.311 | OTI11 | 23 |
| 2011 | OTI | OTILT.11.PC.337 | OTI11 | 24 |
| 2011 | OTI | OTI.11.PC.299 | OTI11 | 26 |
| 2011 | OTI | OTI2.11.PC.302 | OTI11 | 28 |
| 2011 | OTI | OTI2.11.PC.304 | OTI11 | 29 |
| 2011 | OTI | OTI2.11.PC.310 | OTI11 | 29 |
| 2011 | OTI | OTI2.11.PC.290 | OTI11 | 42 |
| 2012 | Fitzroy | F3.12.PC.167 | Fitzroy12 | 14 |
| 2012 | Fitzroy | F4.12.PC.195 | Fitzroy12 | 15 |
| 2012 | Fitzroy | F3.12.PC.160 | Fitzroy12 | 16 |
| 2012 | Fitzroy | F3.12.PC.179 | Fitzroy12 | 16 |
| 2012 | Fitzroy | F3.12.PC.162 | Fitzroy12 | 17 |
| 2012 | Fitzroy | F3.12.PC.175 | Fitzroy12 | 17 |
| 2012 | Fitzroy | F4.12.PC.193 | Fitzroy12 | 17 |
| 2012 | Fitzroy | F3.12.PC.159 | Fitzroy12 | 18 |
| 2012 | Fitzroy | F3.12.PC.166 | Fitzroy12 | 18 |
| 2012 | Fitzroy | F4.12.PC.196 | Fitzroy12 | 18 |
| 2012 | Fitzroy | F3.12.PC.178 | Fitzroy12 | 19 |
| 2012 | Fitzroy | F4.12.PC.191 | Fitzroy12 | 19 |
| 2012 | Fitzroy | F3.12.PC.165 | Fitzroy12 | 20 |
| 2012 | Fitzroy | F3.12.PC.171 | Fitzroy12 | 20 |
| 2012 | Fitzroy | F4.12.PC.194 | Fitzroy12 | 20 |
| 2012 | Fitzroy | F3.12.PC.174 | Fitzroy12 | 21 |
| 2012 | Fitzroy | F3.12.PC.176 | Fitzroy12 | 21 |
| 2012 | Fitzroy | F4.12.PC.191 | Fitzroy12 | 22 |
| 2012 | Fitzroy | F3.12.PC.173 | Fitzroy12 | 23 |
| 2012 | Fitzroy | F3.12.PC.164 | Fitzroy12 | 30 |
| 2012 | Fitzroy | F3.12.PC.168 | Fitzroy12 | 31 |
| 2012 | OTI | OTI3.12.PC.272 | OTI12 | 20 |
| 2012 | OTI | OTI.12.PC.291 | OTI12 | 26 |
| 2012 | OTI | OTI.12.PC.290 | OTI12 | 28 |
| 2012 | OTIt | OTI.12.PC.285 | OTI12 | 23 |
| 2015 | Heron | He1.15.PC.306 | Heron15 | 15 |
| 2015 | Heron | He2.15.PC.315 | Heron15 | 16 |
| 2015 | Heron | He2.15.PC.333 | Heron15 | 16 |
| 2015 | Heron | He1.15.PC.303 | Heron15 | 17 |
| 2015 | Heron | He2.15.PC.328 | Heron15 | 17 |
| 2015 | Heron | He2.15.PC.330 | Heron15 | 17 |
| 2015 | Heron | He2.15.PC.338 | Heron15 | 17 |
| 2015 | Heron | He2.15.PC.319 | Heron15 | 18 |
| 2015 | Heron | He2.15.PC.331 | Heron15 | 18 |
| 2015 | Heron | He1.15.PC.295 | Heron15 | 19 |
| 2015 | Heron | He2.15.PC.322 | Heron15 | 19 |
| 2015 | Heron | He2.15.PC.332 | Heron15 | 19 |
| 2015 | Heron | He2.15.PC.340 | Heron15 | 19 |
| 2015 | Heron | He2.15.PC.314 | Heron15 | 20 |
| 2015 | Heron | He2.15.PC.316 | Heron15 | 20 |
| 2015 | Heron | He1.15.PC.293 | Heron15 | 21 |
| 2015 | Heron | He1.15.PC.297 | Heron15 | 21 |
| 2015 | Heron | He1.15.PC.298 | Heron15 | 21 |
| 2015 | Heron | He1.15.PC.299 | Heron15 | 21 |
| 2015 | Heron | He1.15.PC.302 | Heron15 | 21 |
| 2015 | Heron | He1.15.PC.304 | Heron15 | 21 |
| 2015 | Heron | He1.15.PC.305 | Heron15 | 21 |
| 2015 | Heron | He2.15.PC.318 | Heron15 | 21 |
| 2015 | Heron | He2.15.PC.324 | Heron15 | 21 |
| 2015 | Heron | He2.15.PC.335 | Heron15 | 21 |
| 2015 | Heron | He2.15.PC.336 | Heron15 | 21 |
| 2015 | Heron | He1.15.PC.307 | Heron15 | 22 |
| 2015 | Heron | He2.15.PC.326 | Heron15 | 22 |
| 2015 | Heron | He2.15.PC.337 | Heron15 | 23 |
| 2015 | Heron | He1.15.PC.308 | Heron15 | 24 |
| 2015 | Heron | He2.15.PC.320 | Heron15 | 24 |
| 2015 | Heron | He2.15.PC.321 | Heron15 | 24 |
| 2015 | Heron | He1.15.PC.309 | Heron15 | 25 |
| 2015 | Heron | He2.15.PC.323 | Heron15 | 25 |
| 2015 | Heron | He2.15.PC.329 | Heron15 | 26 |
| 2015 | Heron | He1.15.PC.294 | Heron15 | 27 |
| 2015 | Heron | He2.15.PC.313 | Heron15 | 27 |
| 2015 | Heron | He1.15.PC.296 | Heron15 | 32 |
| 2015 | Heron | He1.15.PC.300 | Heron15 | 32 |
| 2015 | OTI | OTIWi.15.PC.675 | OTI15 | 17 |
| 2015 | OTI | OTIWi.15.PC.667 | OTI15 | 18 |
| 2015 | OTI | OTITt.15.PC.644 | OTI15 | 19 |
| 2015 | OTI | OTITt.15.PC.645 | OTI15 | 19 |
| 2015 | OTI | OTITt.15.PC.648 | OTI15 | 19 |
| 2015 | OTI | OTITt.15.PC.652 | OTI15 | 19 |
| 2015 | OTI | OTIWi.15.PC.655 | OTI15 | 19 |
| 2015 | OTI | OTITt.15.PC.630 | OTI15 | 20 |
| 2015 | OTI | OTITt.15.PC.641 | OTI15 | 20 |
| 2015 | OTI | OTITt.15.PC.643 | OTI15 | 20 |
| 2015 | OTI | OTIWi.15.PC.662 | OTI15 | 20 |
| 2015 | OTI | OTIWi.15.PC.666 | OTI15 | 20 |
| 2015 | OTI | OTITt.15.PC.646 | OTI15 | 21 |
| 2015 | OTI | OTIWi.15.PC.659 | OTI15 | 21 |
| 2015 | OTI | OTIWi.15.PC.661 | OTI15 | 21 |
| 2015 | OTI | OTIWi.15.PC.670 | OTI15 | 21 |
| 2015 | OTI | OTIWi.15.PC.673 | OTI15 | 21 |
| 2015 | OTI | OTITt.15.PC.634 | OTI15 | 22 |
| 2015 | OTI | OTIWi.15.PC.658 | OTI15 | 22 |
| 2015 | OTI | OTIWi.15.PC.676 | OTI15 | 22 |
| 2015 | OTI | OTITt.15.PC.647 | OTI15 | 23 |
| 2015 | OTI | OTIWi.15.PC.660 | OTI15 | 23 |
| 2015 | OTI | OTITt.15.PC.635 | OTI15 | 24 |
| 2015 | OTI | OTIWi.15.PC.664 | OTI15 | 24 |
| 2015 | OTI | OTIWi.15.PC.674 | OTI15 | 24 |
| 2015 | OTI | OTIWi.15.PC.668 | OTI15 | 25 |
| 2015 | OTI | OTIWi.15.PC.671 | OTI15 | 25 |
| 2015 | OTI | OTITt.15.PC.639 | OTI15 | 26 |
| 2015 | OTI | OTITt.15.PC.629 | OTI15 | 27 |
| 2015 | OTI | OTITt.15.PC.636 | OTI15 | 27 |
| 2015 | OTI | OTITt.15.PC.642 | OTI15 | 27 |
| 2015 | OTI | OTIWi.15.PC.669 | OTI15 | 27 |
| 2015 | OTI | OTIWi.15.PC.672 | OTI15 | 27 |
| 2015 | OTI | OTIWi.15.PC.656 | OTI15 | 30 |
| 2015 | OTI | OTITt.15.PC.638 | OTI15 | 31 |
